# Health behavior in Russia during the COVID-19 pandemic

**DOI:** 10.3389/fpubh.2023.1276291

**Published:** 2023-10-02

**Authors:** Anastasia Peshkovskaya, Stanislav Galkin

**Affiliations:** ^1^Tomsk State University, Tomsk, Russia; ^2^Mental Health Research Institute, Tomsk National Research Medical Center, Russian Academy of Sciences, Tomsk, Russia

**Keywords:** pandemic, health behavior, COVID-19, prevention, perceived vulnerability, vaccine hesitancy, conspiracy, public health policy

## Abstract

In this article, we report results from a nationwide survey on pandemic-related health behavior in Russia. A total of 2,771 respondents aged 18 to 82 were interviewed between January 21 and March 3, 2021. The survey included questions on perceived vulnerability to coronavirus, prevention-related health behavior, readiness for vaccination, and general awareness about COVID-19. Descriptive data showed that 21.2% of respondents reported high vulnerability to the coronavirus, and 25% expressed fear. Moreover, 38.7% of the surveyed individuals reported low trust in vaccination efficacy, and 57.5% were unwilling to take a vaccine, which was much higher than the official data. Based on the evidence obtained, four types of health behavior during the pandemic were constructed. Rational (29.3%) and denying (28.6%) behaviors prevailed in men, while women were found to more likely behave with a vaccine-hesitant demeanor (35.7%). Educational background affected the proportion of respondents with the denying type of health behavior, who were also of younger age. The rational behavioral type was found to be more common among respondents aged above 50 years and prevailed as well among individuals with university degrees. The middle-aged population of Russia was highly compliant with prevention-related health practices; however, vaccine hesitancy was also high among them. Furthermore, health behaviors varied significantly across the Federal Districts of Russia. We are convinced that our results contribute to existing public health practices and may help improve communication campaigns to cause positive health behaviors.

## 1. Introduction

The spread of COVID-19 varied significantly over time. The current coronavirus variant of EG.5 was evaluated by the World Health Organization as having low public health risk at the global level (1); however, it has shown increased prevalence, growth advantage, and immune escape properties. As herd immunity rates vary across world regions, epidemiological risks still exist.

Since the first outbreak of COVID-19, individual demographic and socioeconomic characteristics, perceptions of illness, and preventive health behaviors were found to be critical for disease transmission (2–6). Trust in vaccination and, particularly, vaccine hesitancy are also considered highly important (7, 8). Knowledge of relationships between these parameters is fundamental to providing a critical understanding of how experts should best respond to public health challenges. Due to the vaccine skepticism and slower COVID-19 vaccination campaign in Russia reported in the last couple of years compared to most other European countries (9), public policy should consider the dimensions of health behavior to increase disease prevention and vaccination trust.

The aim of this study was to gain an understanding of pandemic-related health behavior in the population of Russia. A range of factors, including basic demographics, educational background, preventive practices, vaccine trust, and conspiracy beliefs, was investigated to plot health behavior determinants during the pandemic.

## 2. Methods

The survey was conducted from 21 January to 3 March 2021 during a period of the second peak incidence of the coronavirus in Russia that had started in late December 2020 when 29,350 infections were registered per day (10). Due to anti-covid restrictions, the study was implemented online by sharing a direct link to an electronic form on social networks. The survey included questions on perceived vulnerability to coronavirus infection, prevention-related behavioral practices (washing hands, wearing a face mask, physical distancing in public places, etc.), COVID-19 vaccination attitude, and coronavirus awareness (general knowledge and conspiracy beliefs) (see Supplementary Table 1).

The survey sample included the full response data of 2,771 participants (66.9% female) aged 16 to 82 (mean age 25.6 ± 10.8 years), who were residents of the Central Federal District of Russia (40%), Northwestern Federal District (10.4%), Volga Federal District (27.3%), Southern Federal District (10.6%), and Siberian Federal District (6.6%). In all, 5.1% of the respondents preferred not to disclose their place of residence, and 729 respondents (26.3%) reported a history of COVID-19 disease. Most of them reported a mild form of the disease (87.8%), while 12.2% indicated a history of severe COVID-19. Detailed participants’ characteristics are presented in Supplementary Table 2.

Participants’ degree of compliance with prevention-related behavioral practices was assessed via Question 8. The answer options consisted of a five-point Likert scale, ranging from “Never” to “Always.” “Never” and “Rarely” responses to any of the Q8 subquestions were encoded as low compliance with preventive practices. Attitudes toward vaccination were investigated via Question 9 and Question 10 based on a three-point Likert scale with answer options ranging from “Disagree” to “Agree.” The response “Disagree” to Q9 or Q10 was weighed as low trust in the COVID-19 vaccine. To allocate complex behavioral types during the pandemic, the data of low vs. high compliance with preventive practices and low vs. high trust to vaccination were aggregated and analyzed.

R software was used to process the data. Measures of frequency and chi-square (*χ*^2^) statistic were applied.

The study was approved by the Ethics Council of Tomsk State University (Approval 101–2020 on 15 December, 2020). All the respondents signed an electronic informed consent form.

## 3. Results

### 3.1. Perceived vulnerability to COVID-19, prevention behavior, and vaccination hesitancy

**Figure 1:**
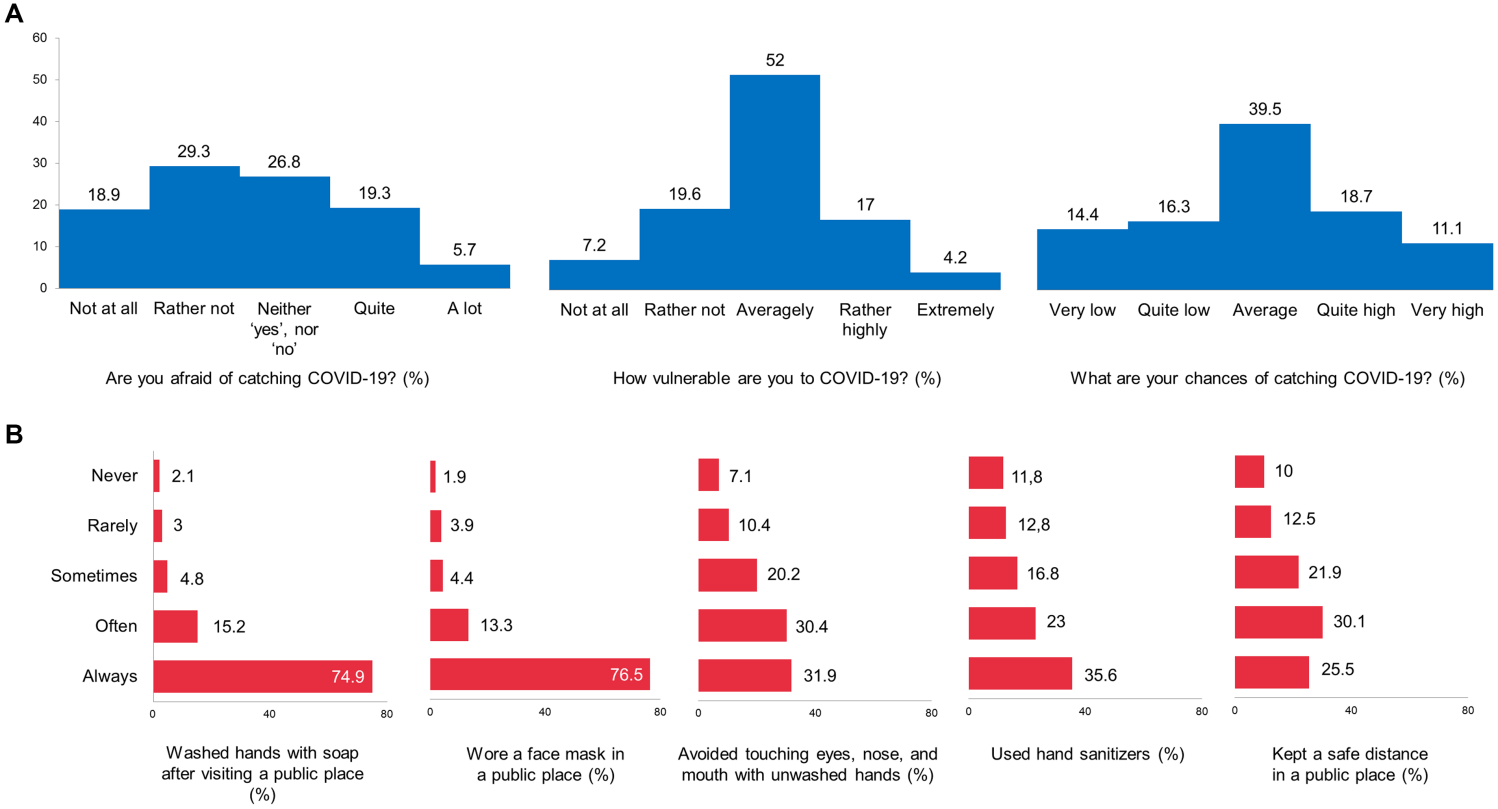
Perceived vulnerability to COVID-19 **(A)** and Compliance with prevention measures **(B)**, the data of the nationwide survey in Russia.

To investigate perceived vulnerability to coronavirus infection, preventive behavior, COVID-19 vaccination attitude, and coronavirus awareness during the second wave of the pandemic in Russia, data on the survey participants’ responses were analyzed. At first, we found that 25% of all the respondents were afraid of catching COVID-19, having answered “A lot” (5.7%) and “Quite afraid” (19.3%) to the question “Are you afraid of catching COVID-19?.” A total of 21.2% of the respondents perceived themselves as vulnerable to the infection by agreeing with the answers “Extremely” (4.2%) and “Rather highly” (17%) to the question “How vulnerable are you to COVID-19?.” In addition, 29.8% of the survey participants reported a relatively high probability of future infection, having answered “Very high” (11.1%) and “Quite high” (18.7%) to the question “What are your chances of catching COVID-19?” Detailed response data are presented in Figure 1A.

Regarding preventive practices, particularly COVID-19 prevention, the majority of the respondents always or at least often followed existing recommendations. They reported washing hands with soap after visiting public places (90.1% in total), wearing a face mask in public places (89.8%), avoiding touching their eyes, nose, and mouth with unwashed hands (62.3%), using hand sanitizers (58.6%), and physical distancing in public places (55.6%) (Figure 1B).

**Figure 2:**
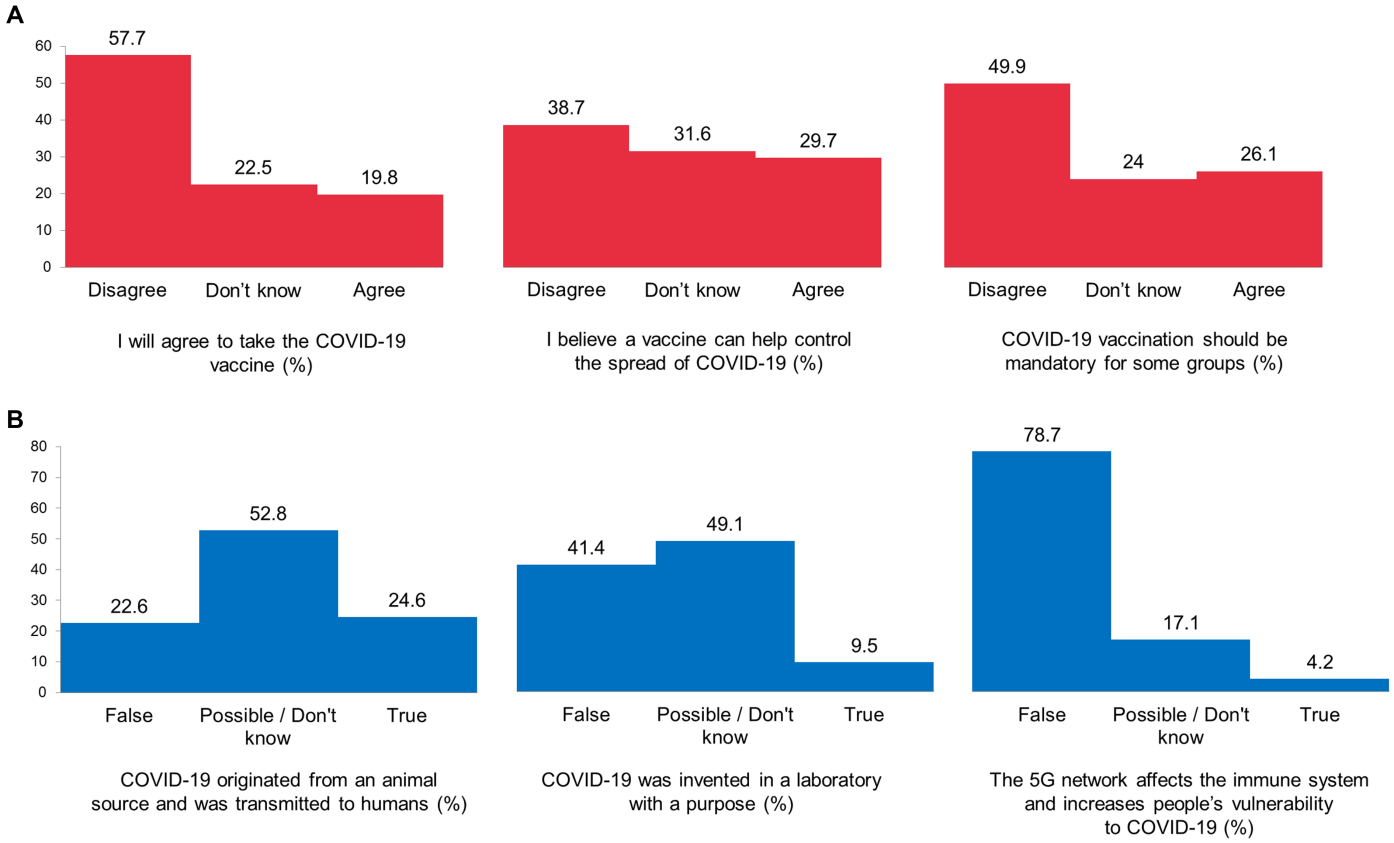
Attitudes towards COVID-19 vaccination **(A)** and COVID-19 conspiracy beliefs **(B)**, the data of the nationwide survey in Russia.

Attitudes toward the COVID-19 vaccine and readiness to vaccinate were of particular interest in this January–March 2021 survey. The survey statement “I will agree to take the COVID-19 vaccine” received 57.7% negative, 19.8% positive, and 22.5% “Do not know” responses. In addition, 38.7% of the respondents disagreed that “a vaccine can help control the spread of COVID-19,” 31.6% said they did not know, and 29.7% of the respondents agreed. The statement “COVID-19 vaccination should be mandatory for some groups” was supported by 26.1% of the respondents, while 49.9% disagreed. Detailed response data are presented in Figure 2A.

Data on coronavirus awareness showed that only 24.6% of the respondents believed coronavirus was of a natural origin. Meanwhile, 9.5% believed that “COVID-19 was invented in a laboratory with a purpose,” and 49.1% of the survey participants considered a laboratory origin of COVID-19 to be possible or found it difficult to answer (Figure 2B).

Based on the data obtained and considering the dimensions of (1) compliance with prevention-related behavioral practices, low vs. high, (2) trust in a COVID-19 vaccine, including readiness to vaccinate, low vs. high, four behavioral types were constructed for further analysis.

Type 1 “Rational”: A total of 753 respondents (27.2%), who reported high compliance with prevention-related behavioral practices and high trust in a COVID-19 vaccine;

Type 2 “Vaccine hesitant”: A total of 892 respondents (32.2%), who reported high compliance with prevention-related behavioral practices but low trust in a COVID-19 vaccine;

Type 3 “Denying”: A total of 762 respondents (27.5%), who reported low compliance with preventive practices and low trust in a COVID-19 vaccine;

Type 4 “Inconsistent”: A total of 364 respondents (13.1%), who reported low compliance with preventive practices but high trust in a COVID-19 vaccine.

On the whole, almost one-third (27.2%) of the surveyed individuals behaved in accordance with recommendations on COVID-19 prevention and were ready to take a vaccine, and another third (27.5%) did not comply with recommendations and showed no trust in preventive practices and vaccination. Another third of the respondents (32.2%) expressed vaccine hesitancy, but they maintained preventive behavior, while 13.1% of the surveyed individuals reported trust in a COVID-19 vaccine but not in COVID-19 preventive practices. Next, we analyzed the demographic differentiations of the behavioral types.

### 3.2. Behavioral types in depth: role of sex, age, education, and COVID-19 conspiracy beliefs

The respondents’ behavioral-type distribution by sex showed that the major part of male respondents comprised “Rational” and “Denying” behavioral types (29.3 and 28.6%, respectively), while the female respondents were found to keep mostly “Vaccine-hesitant” behavior (35.7%), *χ*^2^ (3, *N* = 2,771) = 41.466, *p* < 0.001. The data on distribution are provided in Table 1.

**Table 1:** Distributions of health behaviors by socio-demographic characteristics and conspiracy beliefs (% by line).

Variable	Rational	Vaccine Hesitant	Denying	Inconsistent
Sex, *χ*^2^ (3, *N* = 2,771) = 41.466, *p* < 0.001				
Male	268 (29.3%)	229 (25%)	262 (28.6%)	157 (17.1%)
Female	485 (26.1%)	663 (35.7%)	500 (27%)	207 (11.2%)
Age, *χ*^2^ (15, *N* = 2,771) = 96.008, *p* < 0.001				
under 20	225 (24.4%)	287 (31.2%)	292 (31.7%)	117 (12.7%)
20–29	282 (23.2%)	421 (34.7%)	347 (28.6%)	164 (13.5%)
30–39	91 (32.7%)	86 (31%)	59 (21.2%)	42 (15.1%)
40–49	81 (36.6%)	63 (28.5%)	49 (22.2%)	28 (12.7%)
50–59	52 (60.5%)	21 (24.4%)	6 (7%)	7 (8.1%)
60 and above	22 (43.1%)	14 (27.5%)	9 (17.6%)	6 (11.8%)
Education, *χ*^2^ (12, *N* = 2,771) = 45.917, *p* < 0.001				
Higher education	309 (33%)	290 (31%)	206 (22%)	132 (14%)
Incomplete higher education	286 (23.9%)	406 (33.9%)	351 (29.4%)	153 (12.8%)
Vocational secondary education	35 (18.8%)	63 (33.9%)	62 (33.3%)	26 (14%)
Secondary education	116 (28.1%)	121 (29.4%)	126 (30.6%)	49 (11.9%)
Incomplete secondary education	7 (17.5%)	12 (30%)	16 (40%)	5 (12.5%)
Conspiracy beliefs, *χ*^2^ (3, *N* = 2,771) = 47.635, *p* < 0.001				
Yes	44 (14.5%)	104 (34.2%)	126 (41.5%)	30 (9.8%)
No	709 (28.7%)	788 (32%)	636 (25.8%)	334 (13.5%)

To investigate the age-specific distribution of the behavioral types, we grouped the respondents by their age. We considered age groups of under 20 years (*n* = 921), 20–29 (*n* = 1,214), 30–39 (*n* = 278), 40–49 (*n* = 221), 50–59 (*n* = 86), and 60 years and above (*n* = 51). The results showed that age proportions significantly differed by type, *χ*^2^ (15, *N* = 2,771) = 96.008, *p* < 0.001. A weighty percentage of respondents under the age of 30 showed “Denying” and “Vaccine-hesitant” behavior (60.3 and 65.9% of individuals aged less than 30 years, respectively). They all reported low trust in a COVID-19 vaccine, and the younger they were, the lower compliance was with prevention-related behavioral practices. The age groups of 30–39 and 40–49 showed congruent results in prevention – the vast majority of these middle-aged individuals were highly compliant with preventive recommendations. However, they either reported trust in vaccines (rational type in 32.7 and 36.6% of middle-aged participants, respectively) or had low confidence in vaccination efficacy (vaccine-hesitant behavior in 31 and 28.5% of cases, respectively). At the same time, a significant part of the respondents of older ages (50–59 and 60 years and above) were found to have the “Rational” behavioral type. Most of them (60.5 and 43.1%, respectively) reported compliance with preventive practices and trust in vaccination. Table 1 summarizes the age-related data.

Since education is widely considered as a factor that influences perceptions of ongoing events and corresponding behavior, including social and health behavior (11–14), we analyzed the education-based distribution of the surveyed individuals among the behavioral types. The differences were significant by type, *χ*^2^ (12, *N* = 2,771) = 45.917, *p* < 0.001, and showed that most of the respondents with incomplete secondary education (40%) comprised the “Denying” behavioral type. At the same time, individuals with secondary and vocational secondary education had “Denying” (30.6 and 33.3%) and “Vaccine-hesitant” (29.4 and 33.9%) health behaviors. The hesitant type was also registered in most of the respondents with incomplete higher education (33.9%), while “Rational” health behavior was found to prevail among individuals with higher education (33%). Detailed distribution data are available in Table 1.

Most respondents who believed in COVID-19 conspiracy theories showed “Denying” behavior (41.5%), whereas individuals with no reported conspiracy beliefs were inclined to “Vaccine-hesitant” (32%) and “Rational” (28.7%) behavioral types, *χ*^2^ (3, *N* = 2,771) = 47.635, *p* < 0.001 (see Table 1).

### 3.3. Regions and health behavior: mapping general trends

Regional data on health behavior prevalence during the second wave of the pandemic in Russia showed that significant differences existed across Federal Districts, *χ*^2^ (15, *N* = 2,771) = 69.26, *p* < 0.001 (Table 2). The vast majority of respondents who resided in the Central Federal District showed “Vaccine-hesitant” (36.6%) and “Rational” (30%) health behavior. Most participants from the Volga Federal District belonged to the “Denying” (34.2%) and “Vaccine-hesitant” (30.4%) behavioral types, while residents of the Siberian Federal District showed “Rational” health behavior more often (29.3%). Two of the largest respondents’ groups from the Northwestern Federal District were found to behave according to the “Rational” (29%) and “Vaccine-hesitant” (28.7%) types. Finally, the surveyed individuals from the Southern Federal District were more differentiated and showed “Vaccine-hesitant” (29.6%), “Rational” (27.7%), and “Denying” behaviors (27.7%) during the reported period of the pandemic.

**Table 2:** Distributions of health behaviors by region (% by line), *χ*^2^ (15, *N* = 2,771) = 69.26, *p* < 0.001.

Residence	Rational	Vaccine Hesitant	Denying	Inconsistent
Central Federal District	333 (30%)	406 (36.6%)	260 (23.4%)	110 (10%)
Northwestern Federal District	84 (29%)	83 (28.7%)	75 (26%)	47 (16.3%)
Volga Federal District	161 (21.3%)	230 (30.4%)	259 (34.2%)	107 (14.1%)
Southern Federal District	81 (27.7%)	87 (29.6%)	81 (27.7%)	44 (15%)
Siberian Federal District	53 (29.3%)	42 (23.2%)	47 (26%)	39 (21.5%)
Undisclosed	41 (28.9%)	44 (31%)	40 (28.1%)	17 (12%)

## 4. Discussion

Obviously, a key challenge for health authorities across the world is to encourage people to accept vaccines. The rates of vaccination skepticism we found in Russia were rather high. The majority of respondents (57.7%) in our study disagreed to take a COVID-19 vaccine, and 38.7% also disagreed that a vaccine can help control the spread of the coronavirus. Interestingly, nine months later, the available data from November 2021 reported by the Russian Public Opinion Research Center via a telephone-based survey methodology (*n* = 1,600) showed that only 32% of the surveyed participants expressed a negative attitude toward vaccination, only 4% did not want to take a vaccine, and 37% of respondents declared that they have already been vaccinated or found it difficult to answer the question (15). Such a gap in figures may be explained both by the difference in methodology, with possible communication-related self-report bias during the telephone interview, and by a positive dynamic in public opinions and vaccine acceptance by the later time period of November 2021. However, even a 32% share of those who perceived vaccination negatively is a risk factor for public health. Existing strategies should be improved to allow vaccination to be understood and accepted as a social practice.

Conspiracy theories about coronavirus and the pandemic are widespread around the world. For example, a survey conducted in the United States (*n* = 2,023) showed that more than 31% agreed that coronavirus was intentionally created and spread (16). The data obtained in our study showed that 49.1% of the respondents considered a laboratory origin of the coronavirus possible or found it difficult to answer, while 9.5% were convinced that laboratory invention of the coronavirus was true. As beliefs in specific conspiracy theories related to the coronavirus are considered among factors negatively affecting the public acceptance of COVID-19 vaccines (17), a high rate of vaccine skepticism registered in Russia may be at least partially explained by the misinformation effect of conspiracy speculations.

Based on the survey evidence on preventive practices and vaccine trust, we allocated four types of health behavior prevalent in Russia during the COVID-19 pandemic and followed their sex-, age-, and education-related specific distributions. The “Rational” and “Denying” behavioral types prevailed in 29.3 and 28.6% of the male subsample, while the female respondents were found to more likely behave in accordance with a vaccine-hesitant demeanor (35.7% of the subsample). This corresponds to the well-described gender differences in behavior (18–20) and the known demographic determinants of health (21, 22), which indicate the greater vulnerability of women to behavioral hesitancy, anxiety, and fear.

The highest rate of COVID-19 conspiracy beliefs (41.5%) was registered among the respondents with “Denying” health behavior, which corresponds, to a certain extent, to the opinions and behaviors interrelation model (23, 24).

Along with that, educational background was found to affect the proportions of respondents with “Rational” and “Denying” behavioral types by doubling the rate of the former from 17.5% among respondents with incomplete secondary education to 33% among individuals with university degrees and by decreasing the rate of the latter from 40 to 22%. “Denying” individuals were also younger (less than 30 years), while “Rational” were older (50 years and above), as older age was and still is a pandemic-related risk factor for heath. The middle-aged population of Russia (30–39 and 40–49 years of age) was highly compliant with prevention-related health practices; however, there were also high rates of vaccine-hesitant behavior among them. As the middle-aged population is most economically active, they should be considered for special targeting when planning a prevention campaign and vaccination promotion.

Despite the significant differences in health behaviors that we found across the Federal Districts of Russia, this study was not aimed to comprehensively address regional and cross-regional tendencies. Given the great variability of environmental factors, social capital, cultural health beliefs, and pandemic-related public health policies among the regional units within the Federal Districts, further research is needed to understand the dimensions of health behavior at a regional level.

As the national healthcare agenda is focused on pandemic-related somatic burden (25), existing comorbidities (26), and mental health risks (27), the evidence reported in our study will invigorate knowledge consolidation for a prompt response to potential infection outbreaks and future public health challenges.

## 5. Conclusion

Our findings contribute to the existing knowledge of health behavior and its determinants. Due to vaccine distrust among the Russian population and the country’s slower vaccination campaign compared to most other European countries during the pandemic, the results we have reported may improve disease prevention and advance communication campaigns to cause positive health behaviors.

## Data availability statement

The datasets presented in this article are not readily available because of ethical restrictions. Requests to access the datasets should be directed to AP, peshkovskaya@gmail.com.

## Ethics statement

The studies involving humans were approved by the Ethics Council of Tomsk State University. The studies were conducted in accordance with the local legislation and institutional requirements. The participants provided their written informed consent to participate in this study.

## Author contributions

AP: Conceptualization, Funding acquisition, Methodology, Validation, Project administration, Resources, Supervision, Writing – original draft, Writing – review & editing. SG: Data curation, Formal analysis, Investigation, Methodology, Writing – original draft.

## Funding

The author(s) declare financial support was received for the research, authorship, and/or publication of this article. This work was supported by Grant no. 075-15-2022-1152 (Resolution no. 619 of April 8, 2022).

## Conflict of interest

The authors declare that the research was conducted in the absence of any commercial or financial relationships that could be construed as a potential conflict of interest.

## Publisher’s note

All claims expressed in this article are solely those of the authors and do not necessarily represent those of their affiliated organizations, or those of the publisher, the editors and the reviewers. Any product that may be evaluated in this article, or claim that may be made by its manufacturer, is not guaranteed or endorsed by the publisher.
