# Supplementary material for: Health behavior in Russia during the COVID-19 pandemic
Source: Front Public Health. 2023 Oct 2;11:1276291. doi: 10.3389/fpubh.2023.1276291 (PMC10577229; doi:10.3389/fpubh.2023.1276291)
Supplement: Supplementary file 1 [file Table_1.docx]

Supplementary Material

Health Behavior in Russia during the COVID-19 Pandemic

Anastasia Peshkovskaya*, Stanislav Galkin

*** Correspondence:** Anastasia Peshkovskaya, peshkovskaya@gmail.com

# Supplementary Tables

Supplementary Table 1

Survey questions

| No. | Question | Type |
| --- | --- | --- |
| Q1 | Please indicate your age | Identification |
| Q2 | Please indicate your sex | Identification |
| Q3 | Please indicate your education | Identification |
| Q4 | Please indicate region of your residence | Identification |
| Q5 | Have you had coronavirus disease (COVID-19)? | Dichotomous scale |
| Q6 | If “Yes” to Q5: How severe was your illness from COVID-19? | Dichotomous scale |
| Q7 | Are you afraid of catching COVID-19? | Rating scale |
| Q8 | How vulnerable are you to COVID-19? | Rating scale |
| Q9 | What are your chances of catching COVID-19? | Rating scale |
| Q10 | During the past 14 days, which of the following measures have you taken to prevent COVID-19 exposure?   - Washed hands with soap after visiting a public place - Wore a face mask in a public place - Avoided touching eyes, nose, and mouth with unwashed hands - Used hand sanitizers - Kept a safe distance in a public place | Rating scale |
| Q11 | I will agree to take the COVID-19 vaccine | Rating scale |
| Q12 | I believe a vaccine can help control the spread of COVID-19 | Rating scale |
| Q13 | COVID-19 vaccination should be mandatory for some groups | Rating scale |
| Q14 | COVID-19 originated from an animal source and was transmitted to humans | Rating scale |
| Q15 | COVID-19 was invented in a laboratory with a purpose | Rating scale |
| Q16 | The 5G network affects the immune system and increases people’s vulnerability to COVID-19 | Rating scale |

Supplementary Table 2

Participants’ characteristics

| Variable | n | % |
| --- | --- | --- |
| Sex | | |
| Males | 916 | 33.1 |
| Females | 1,855 | 66.9 |
| Age | | |
| under 20 | 921 | 33.2 |
| 20–29 | 1,214 | 43.8 |
| 30–39 | 278 | 10 |
| 40–49 | 221 | 8 |
| 50–59 | 86 | 3.1 |
| 60 and above | 51 | 1.8 |
| Education | | |
| Higher education | 937 | 33.8 |
| Incomplete higher education | 1,196 | 43.2 |
| Vocational secondary education | 186 | 6.7 |
| Secondary education | 412 | 14.9 |
| Incomplete secondary education | 40 | 1.4 |
| Residence, Federal District | | |
| Central Federal District | 1,109 | 40 |
| Northwestern Federal District | 289 | 10.4 |
| Volga Federal District | 757 | 27.3 |
| Southern Federal District | 293 | 10.6 |
| Siberian Federal District | 181 | 6.6 |
| Undisclosed | 142 | 5.1 |
| History of COVID-19 | | |
| No | 2,042 | 73.7 |
| Yes | 729 | 26.3 |
| Severity of COVID-19 | | |
| Mild | 640 | 87.8 |
| Severe | 89 | 12.2 |
